# Diaqua­bis(2-pyridylphospho­nato *N*-oxide-κ^2^ *O* ^1^,*O* ^2^)cobalt(II)

**DOI:** 10.1107/S1600536808037185

**Published:** 2008-11-13

**Authors:** Yun-Sheng Ma, Tian-Xian Lu

**Affiliations:** a Department of Chemistry & Materials Engineering, Changshu Institute of Technology, Changshu, 215500 Jiangsu, People’s Republic of China

## Abstract

In the title complex, [Co(C_5_H_5_NO_4_P)_2_(H_2_O)_2_], the Co^II^ ion, which lies on a crystallographic inversion center, is coordin­ated by four O atoms from two bidentate 2-phospho­nato­pyridine *N*-oxide ligands and two O atoms from two water ligands in a slightly distorted octa­hedral environment. Mol­ecules are inter­linked by three O—H⋯O hydrogen bonds and one weak C—H⋯O inter­action, forming a three-dimensional supra­molecular structure.

## Related literature

For new open frameworks based on metal pyridylphospho­nates, see: Ayyappan *et al.* (2001 ▶). For two-dimensional Cu-phospho­nates, see: Ma *et al.* (2006 ▶). For one-dimensional Cu-phospho­nates containing bridging ligands, see: Ma *et al.* (2007 ▶). For catalytic and magnetic properties of metal phospho­nates, see: Cao *et al.* (1992 ▶). For the layered structures of monophospho­nic acids and transition metal ions, see Clearfield (1998 ▶). For a tetra­aqua-Co(II)-4-hydroxy­pyridine-2,6-dicarboxyl­ate structure, see: Cui *et al.* (2006 ▶). For weak C—H⋯O hydrogen-bonding contacts, see: Desiraju & Steiner (2001 ▶). For the synthesis of the ligand (2-pyridyl-*N*-oxide)phospho­nic acid, see: McCabe *et al.* (1987 ▶).
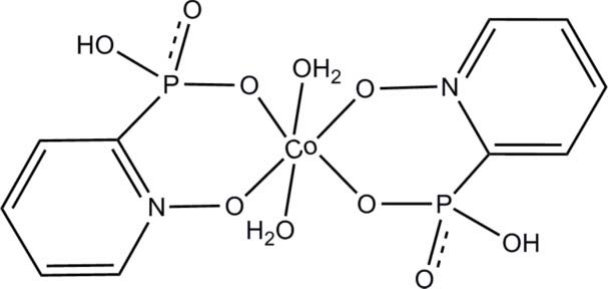


## Experimental

### 

#### Crystal data

[Co(C_5_H_5_NO_4_P)_2_(H_2_O)_2_]
*M* *_r_* = 443.10
Monoclinic, 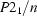

*a* = 4.7899 (10) Å
*b* = 12.075 (2) Å
*c* = 14.162 (3) Å
β = 99.51 (3)°
*V* = 807.8 (3) Å^3^
*Z* = 2
Mo *K*α radiation
μ = 1.32 mm^−1^
*T* = 293 (2) K
0.5 × 0.3 × 0.2 mm

#### Data collection

Rigaku SCX mini diffractometer
Absorption correction: multi-scan (*CrystalClear*; Rigaku, 2005 ▶) *T*_min_ = 0.625, *T*_max_ = 0.766
8068 measured reflections
1848 independent reflections
1373 reflections with *I* > 2σ(*I*)
*R*_int_ = 0.083

#### Refinement

*R*[*F* ^2^ > 2σ(*F* ^2^)] = 0.051
*wR*(*F* ^2^) = 0.109
*S* = 1.05
1848 reflections
127 parameters
H atoms treated by a mixture of independent and constrained refinement
Δρ_max_ = 0.39 e Å^−3^
Δρ_min_ = −0.45 e Å^−3^

### 

Data collection: *CrystalClear* (Rigaku, 2005 ▶); cell refinement: *CrystalClear*; data reduction: *CrystalClear*; program(s) used to solve structure: *SHELXS97* (Sheldrick, 2008 ▶); program(s) used to refine structure: *SHELXL97* (Sheldrick, 2008 ▶); molecular graphics: *SHELXTL/PC* (Sheldrick, 2008 ▶); software used to prepare material for publication: *SHELXTL/PC* and *PLATON* (Spek, 2003 ▶).

## Supplementary Material

Crystal structure: contains datablocks I, global. DOI: 10.1107/S1600536808037185/si2126sup1.cif

Structure factors: contains datablocks I. DOI: 10.1107/S1600536808037185/si2126Isup2.hkl

Additional supplementary materials:  crystallographic information; 3D view; checkCIF report

## Figures and Tables

**Table:** 

Co1—O1	2.084 (2)
Co1—O1*W*	2.099 (3)
Co1—O4	2.131 (2)

**Table:** 

O1—Co1—O1*W*	89.93 (11)
O1—Co1—O4	87.91 (9)
O1*W*—Co1—O4	91.55 (10)

**Table 2:** Hydrogen-bond geometry (Å, °)

*D*—H⋯*A*	*D*—H	H⋯*A*	*D*⋯*A*	*D*—H⋯*A*
O1*W*—H1*WB*⋯O1^i^	0.77 (4)	2.15 (5)	2.803 (4)	142 (4)
O3—H3*A*⋯O2^ii^	0.85 (4)	1.68 (4)	2.516 (3)	171 (4)
O1*W*—H1*WA*⋯O4^ii^	0.77 (4)	2.00 (4)	2.719 (4)	155 (4)
C3—H3⋯O2^iii^	0.93	2.52	3.449 (5)	178

Symmetry codes: (i) 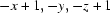; (ii) 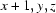; (iii) 
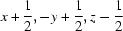
.

**Fig. 1.**
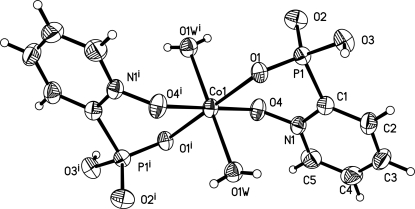
A view of the compound with the atomic numbering scheme. Displacement ellipsoids were drawn at the 40% probability level. [Symmetry code i = (-x, -y, -z + 1)]

**Fig. 2.**
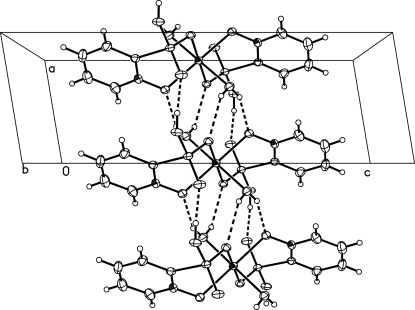
One-dimensional supramolecular chain structure with classic O—H···O hydrogen bonds shown with dashed lines, running along the a axis.

**Fig. 3.**
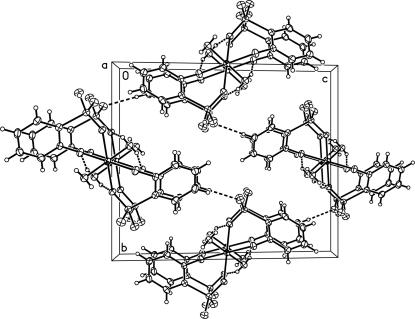
The cell packing diagram indicating weak C—H···O links with dashed lines, viewed down the a axis.

## supplementary crystallographic information

### Comment

The chemistry of metal phosphonates has received an increasing attention in recent years for their new architectures and properties in catalysis, ion exchange and magnetic materials (Cao *et al.*, 1992). It has been well known that the monophosphonic acid *R*PO_3_H_2_, where *R* represents an alkyl or aryl group, prefer to form layered structures with transition metal ions (Clearfield 1998). There are some metal phosphonates reported during the past several years which contain pyridyl groups (Ayyappan *et al.* (2001); Ma *et al.* (2006); Ma *et al.* (2007). The present paper is concerned with the crystal structure of a new cobalt phosphonate complex with a (2-pyridyl-*N*-oxide)phosphonate ligand.

The asymmetric unit contains half of the [Co(C_5_H_4_NOPO_3_H)_2_(H_2_O)_2_] molecule. As shown in Fig. 1, atom Co1 lies on an inversion centre and is coordinated by four O atoms [O1, O1^i^, O4 and O4^i^] from two ligands, and two O atoms from two aqua ligands, thereby forming a slightly distorted CoO_6_ octahedral coordination geometry. The Co1—O1 and the Co1—O4 distances (Table 1) are close to the value observed in [Co*L*_2_(H_2_O)_4_] [2.0653 (12) Å, and the Co—O(H_2_O) distance in the title structure is close to the value observed in the Co-tetraaqua compound [2.0764 (13) Å with *L* = 4-hydroxypyridine-2,6-dicarboxylate (Cui *et al.*, 2006)] (Table 1). The *cisoid* angles of CoO_6_ (Table 1) are close to 90°. The phosphonate serves as a chelating ligand using one pyridyl N-oxide acceptor O atom and one phosphonate oxygen atom. One phosphonate oxygen is protonated with the P1—O3 distance 1.560 (3) Å.

In the complex, three classic intermolecular O—H···O hydrogen-bonds exist between the water molecule and a phosphonate O atom, the water molecule and a pyridyl N-oxide acceptor O atom, and between two phosphonate O atoms (Fig. 1, Table 2). Thus, the molecules are interlinked by these hydrogen bonds, forming a one-dimensional chain structure along the *a*-axis (Fig.2). Additionally, weak intermolecular C—H···O hydrogen bonding contacts (Desiraju & Steiner, 2001) link these chains to form a three-dimensional supramolecular network (Fig. 3).

### Experimental

The synthesis of the ligand (2-pyridyl-*N*-oxide)phosphonic acid, see: McCabe *et al.* (1987). The (2-pyridyl-*N*-oxide)phosphonic acid (0.0176 g, 0.1 mmol) was dissolved in distilled water (5 ml), and was added a solution of Co(NO_3_)_2_^.^6H_2_O (0.0240 g, 0.01 mmol) in distilled water (2 ml). The mixture was stirred at room temperature for 5 h and then filtered. Slow evaporation of the solvent gave pink crystals. (Yield 45%).

### Refinement

Carbon-bound H atoms were positioned geometrically (C—H = 0.93 Å), and were included in the refinement in the riding mode approximation, with *U*_iso_(H) = 1.2*U*_eq_(C). The water H atoms and P—O—H H atom were located in a difference Fourier map and restrained to 0.77 (4) Å and 0.85 (4) Å, respectively, with *U*_iso_(H) refined between 1.0 and 1.8*U*_eq_(O).

### Crystal data

**Table:**

[Co(C_5_H_5_NO_4_P)_2_(H_2_O)_2_]	*F*_000_ = 450
*M**_r_* = 443.10	*D*_x_ = 1.822 Mg m^−^^3^
Monoclinic, *P*2_1_/*n*	Mo *K*α radiation λ = 0.71073 Å
Hall symbol: -P 2yn	Cell parameters from 6955 reflections
*a* = 4.7899 (10) Å	θ = 3.4–27.7º
*b* = 12.075 (2) Å	µ = 1.32 mm^−^^1^
*c* = 14.162 (3) Å	*T* = 293 (2) K
β = 99.51 (3)º	Needle, pink
*V* = 807.8 (3) Å^3^	0.5 × 0.3 × 0.2 mm
*Z* = 2	

### Data collection

**Table:**

Rigaku MACHINE? diffractometer	1848 independent reflections
Radiation source: fine-focus sealed tube	1373 reflections with *I* > 2σ(*I*)
Monochromator: graphite	*R*_int_ = 0.083
Detector resolution: 13.6612 pixels mm^-1^	θ_max_ = 27.5º
*T* = 293(2) K	θ_min_ = 3.4º
dtfind.ref scans	*h* = −6→6
Absorption correction: multi-scan(CrystalClear; Rigaku, 2005)	*k* = −15→15
*T*_min_ = 0.625, *T*_max_ = 0.766	*l* = −18→18
8068 measured reflections	

### Refinement

**Table:**

Refinement on *F*^2^	Secondary atom site location: difference Fourier map
Least-squares matrix: full	Hydrogen site location: inferred from neighbouring sites
*R*[*F*^2^ > 2σ(*F*^2^)] = 0.051	H atoms treated by a mixture of independent and constrained refinement
*wR*(*F*^2^) = 0.109	*w* = 1/[σ^2^(*F*_o_^2^) + (0.0457*P*)^2^ + 0.1404*P*] where *P* = (*F*_o_^2^ + 2*F*_c_^2^)/3
*S* = 1.05	(Δ/σ)_max_ < 0.001
1848 reflections	Δρ_max_ = 0.39 e Å^−^^3^
127 parameters	Δρ_min_ = −0.45 e Å^−^^3^
Primary atom site location: structure-invariant direct methods	Extinction correction: none

### Special details

**Table:**

Geometry. All e.s.d.'s (except the e.s.d. in the dihedral angle between two l.s. planes) are estimated using the full covariance matrix. The cell e.s.d.'s are taken into account individually in the estimation of e.s.d.'s in distances, angles and torsion angles; correlations between e.s.d.'s in cell parameters are only used when they are defined by crystal symmetry. An approximate (isotropic) treatment of cell e.s.d.'s is used for estimating e.s.d.'s involving l.s. planes.
Refinement. Refinement of *F*^2^ against ALL reflections. The weighted *R*-factor *wR* and goodness of fit *S* are based on *F*^2^, conventional *R*-factors *R* are based on *F*, with *F* set to zero for negative *F*^2^. The threshold expression of *F*^2^ > σ(*F*^2^) is used only for calculating *R*-factors(gt) *etc*. and is not relevant to the choice of reflections for refinement. *R*-factors based on *F*^2^ are statistically about twice as large as those based on *F*, and *R*- factors based on ALL data will be even larger.

### Fractional atomic coordinates and isotropic or equivalent isotropic displacement parameters (Å^2^)

**Table:**

	*x*	*y*	*z*	*U*_iso_*/*U*_eq_	
Co1	0.0000	0.0000	0.5000	0.0251 (2)	
P1	0.06589 (18)	0.23457 (7)	0.41631 (6)	0.0247 (2)	
O1	0.2057 (5)	0.14984 (17)	0.48661 (15)	0.0265 (5)	
O2	−0.1949 (5)	0.2903 (2)	0.43729 (17)	0.0367 (6)	
O3	0.2811 (5)	0.3247 (2)	0.39564 (18)	0.0328 (6)	
H3A	0.455 (9)	0.307 (3)	0.406 (3)	0.055 (14)*	
O4	−0.2925 (5)	0.0442 (2)	0.37548 (16)	0.0326 (6)	
O1W	0.2669 (6)	−0.0782 (3)	0.4170 (2)	0.0317 (6)	
H1WA	0.362 (8)	−0.043 (3)	0.389 (3)	0.031 (12)*	
H1WB	0.362 (9)	−0.120 (4)	0.449 (3)	0.057 (17)*	
N1	−0.1928 (6)	0.0750 (2)	0.29606 (19)	0.0281 (7)	
C1	−0.0157 (7)	0.1641 (3)	0.3010 (2)	0.0273 (8)	
C2	0.0908 (8)	0.1928 (3)	0.2193 (3)	0.0365 (9)	
H2	0.2141	0.2525	0.2210	0.044*	
C3	0.0168 (9)	0.1342 (3)	0.1355 (3)	0.0450 (10)	
H3	0.0910	0.1534	0.0811	0.054*	
C4	−0.1695 (9)	0.0462 (4)	0.1332 (3)	0.0465 (11)	
H4	−0.2257	0.0071	0.0767	0.056*	
C5	−0.2703 (9)	0.0173 (3)	0.2146 (3)	0.0407 (10)	
H5	−0.3930	−0.0426	0.2138	0.049*	

### Atomic displacement parameters (Å^2^)

**Table:**

	*U*^11^	*U*^22^	*U*^33^	*U*^12^	*U*^13^	*U*^23^
Co1	0.0192 (4)	0.0303 (4)	0.0261 (4)	−0.0024 (3)	0.0050 (3)	0.0041 (3)
P1	0.0175 (5)	0.0272 (5)	0.0299 (5)	−0.0009 (4)	0.0049 (4)	0.0017 (4)
O1	0.0208 (12)	0.0281 (12)	0.0301 (13)	−0.0053 (10)	0.0031 (10)	0.0049 (10)
O2	0.0216 (14)	0.0389 (15)	0.0494 (16)	0.0026 (12)	0.0056 (12)	−0.0042 (12)
O3	0.0177 (14)	0.0330 (14)	0.0464 (16)	−0.0028 (12)	0.0010 (12)	0.0074 (11)
O4	0.0244 (14)	0.0436 (15)	0.0301 (13)	−0.0085 (11)	0.0050 (11)	0.0067 (11)
O1W	0.0271 (15)	0.0354 (16)	0.0339 (16)	−0.0008 (14)	0.0084 (13)	0.0033 (13)
N1	0.0279 (16)	0.0325 (16)	0.0232 (15)	0.0009 (14)	0.0020 (12)	0.0032 (12)
C1	0.0207 (18)	0.0320 (19)	0.0288 (18)	0.0021 (16)	0.0033 (15)	0.0034 (15)
C2	0.035 (2)	0.040 (2)	0.037 (2)	0.0009 (18)	0.0113 (18)	0.0096 (17)
C3	0.056 (3)	0.053 (3)	0.028 (2)	0.011 (2)	0.0147 (19)	0.0060 (18)
C4	0.062 (3)	0.044 (2)	0.031 (2)	0.010 (2)	0.002 (2)	−0.0053 (18)
C5	0.046 (3)	0.037 (2)	0.037 (2)	−0.0031 (19)	−0.0016 (19)	−0.0040 (17)

### Geometric parameters (Å, °)

**Table:**

Co1—O1	2.084 (2)	O1W—H1WA	0.77 (4)
Co1—O1^i^	2.084 (2)	O1W—H1WB	0.77 (4)
Co1—O1W^i^	2.099 (3)	N1—C5	1.346 (4)
Co1—O1W	2.099 (3)	N1—C1	1.365 (4)
Co1—O4	2.131 (2)	C1—C2	1.383 (5)
Co1—O4^i^	2.131 (2)	C2—C3	1.377 (5)
P1—O2	1.491 (2)	C2—H2	0.9300
P1—O1	1.505 (2)	C3—C4	1.384 (6)
P1—O3	1.560 (3)	C3—H3	0.9300
P1—C1	1.826 (3)	C4—C5	1.367 (5)
O3—H3A	0.85 (4)	C4—H4	0.9300
O4—N1	1.345 (3)	C5—H5	0.9300
			
O1—Co1—O1^i^	180.0	N1—O4—Co1	119.04 (18)
O1—Co1—O1W^i^	90.07 (11)	Co1—O1W—H1WA	120 (3)
O1^i^—Co1—O1W^i^	89.93 (11)	Co1—O1W—H1WB	108 (3)
O1—Co1—O1W	89.93 (11)	H1WA—O1W—H1WB	108 (4)
O1^i^—Co1—O1W	90.07 (11)	O4—N1—C5	119.2 (3)
O1W^i^—Co1—O1W	180.000 (1)	O4—N1—C1	118.6 (3)
O1—Co1—O4	87.91 (9)	C5—N1—C1	122.2 (3)
O1^i^—Co1—O4	92.09 (9)	N1—C1—C2	117.8 (3)
O1W^i^—Co1—O4	88.45 (10)	N1—C1—P1	116.9 (2)
O1W—Co1—O4	91.55 (10)	C2—C1—P1	125.3 (3)
O1—Co1—O4^i^	92.09 (9)	C3—C2—C1	120.9 (4)
O1^i^—Co1—O4^i^	87.91 (9)	C3—C2—H2	119.5
O1W^i^—Co1—O4^i^	91.55 (10)	C1—C2—H2	119.5
O1W—Co1—O4^i^	88.45 (10)	C2—C3—C4	119.2 (4)
O4—Co1—O4^i^	180.00 (9)	C2—C3—H3	120.4
O2—P1—O1	118.07 (14)	C4—C3—H3	120.4
O2—P1—O3	108.89 (15)	C5—C4—C3	119.5 (4)
O1—P1—O3	111.28 (14)	C5—C4—H4	120.2
O2—P1—C1	109.02 (15)	C3—C4—H4	120.2
O1—P1—C1	106.32 (14)	N1—C5—C4	120.3 (4)
O3—P1—C1	101.99 (15)	N1—C5—H5	119.9
P1—O1—Co1	119.01 (13)	C4—C5—H5	119.9
P1—O3—H3A	117 (3)		
			
O2—P1—O1—Co1	68.83 (18)	O4—N1—C1—P1	−0.7 (4)
O3—P1—O1—Co1	−164.20 (13)	C5—N1—C1—P1	179.3 (3)
C1—P1—O1—Co1	−53.93 (18)	O2—P1—C1—N1	−68.0 (3)
O1W^i^—Co1—O1—P1	−78.04 (16)	O1—P1—C1—N1	60.3 (3)
O1W—Co1—O1—P1	101.96 (16)	O3—P1—C1—N1	176.9 (2)
O4—Co1—O1—P1	10.41 (15)	O2—P1—C1—C2	113.0 (3)
O4^i^—Co1—O1—P1	−169.59 (15)	O1—P1—C1—C2	−118.7 (3)
O1—Co1—O4—N1	52.1 (2)	O3—P1—C1—C2	−2.0 (3)
O1^i^—Co1—O4—N1	−127.9 (2)	N1—C1—C2—C3	0.9 (5)
O1W^i^—Co1—O4—N1	142.2 (2)	P1—C1—C2—C3	179.9 (3)
O1W—Co1—O4—N1	−37.8 (2)	C1—C2—C3—C4	0.8 (6)
Co1—O4—N1—C5	121.0 (3)	C2—C3—C4—C5	−1.8 (6)
Co1—O4—N1—C1	−59.0 (3)	O4—N1—C5—C4	−179.3 (3)
O4—N1—C1—C2	178.3 (3)	C1—N1—C5—C4	0.7 (5)
C5—N1—C1—C2	−1.7 (5)	C3—C4—C5—N1	1.1 (6)

Symmetry codes: (i) −*x*, −*y*, −*z*+1.

### Hydrogen-bond geometry (Å, °)

**Table:**

*D*—H···*A*	*D*—H	H···*A*	*D*···*A*	*D*—H···*A*
O1W—H1WB···O1^ii^	0.77 (4)	2.15 (5)	2.803 (4)	142 (4)
O3—H3A···O2^iii^	0.85 (4)	1.68 (4)	2.516 (3)	171 (4)
O1W—H1WA···O4^iii^	0.77 (4)	2.00 (4)	2.719 (4)	155 (4)
C3—H3···O2^iv^	0.93	2.52	3.449 (5)	178

Symmetry codes: (ii) −*x*+1, −*y*, −*z*+1; (iii) *x*+1, *y*, *z*; (iv) *x*+1/2, −*y*+1/2, *z*−1/2.

## References

[bb1] Ayyappan, P., Evans, O. R., Foxman, B. M., Wheeler, K. A., Warren, T. H. & Lin, W.-B. (2001). *Inorg. Chem.* **40**, 5954–5961. 10.1021/ic010609g 11681911

[bb2] Cao, G., Hong, H. & Mallouk, T. E. (1992). *Acc. Chem. Res.* **25**, 420–427.

[bb3] Clearfield, A. (1998). *Prog. Inorg. Chem.* **47**, 371–510.

[bb4] Cui, J.-Z., Zhang, H., Lin, T., Kang, H.-J. & Gao, H.-L. (2006). *Acta Cryst.* E**62**, m2499–m2501.

[bb5] Desiraju, G. R. & Steiner, T. (2001). *The Weak Hydrogen Bond in Structural Chemistry and Biology*, pp. 29–121. IUCr Monograph on Crystallography, No. 9. Oxford University Press.

[bb6] Ma, Y.-S., Song, Y., Du, W.-X., Li, Y.-Z. & Zheng, L.-M. (2006). *Dalton Trans.* pp. 3228–3235. 10.1039/b517311f 16802041

[bb7] Ma, Y.-S., Wang, T.-W., Li, Y.-Z. & Zheng, L.-M. (2007). *Inorg. Chim. Acta*, **360**, 4117–4124.

[bb8] McCabe, D. J., Russell, A. A., Karthikeyan, S., Paine, R. T., Ryan, R. R. & Smith, B. (1987). *Inorg. Chem.* **26**, 1230–1235.

[bb9] Rigaku (2005). *CrystalClear* Rigaku Corporation, Tokyo, Japan.

[bb10] Sheldrick, G. M. (2008). *Acta Cryst.* A**64**, 112–122. 10.1107/S0108767307043930 18156677

[bb11] Spek, A. L. (2003). *J. Appl. Cryst.* **36**, 7–13.

